# Long-Read Genome Assembly of Saccharomyces uvarum Strain CBS 7001

**DOI:** 10.1128/mra.00972-21

**Published:** 2022-01-06

**Authors:** Jingxuan Chen, David J. Garfinkel, Casey M. Bergman

**Affiliations:** a Institute of Bioinformatics, University of Georgia, Athens, Georgia, USA; b Department of Genetics, University of Georgia, Athens, Georgia, USA; c Department of Biochemistry & Molecular Biology, University of Georgia, Athens, Georgia, USA; Vanderbilt University

## Abstract

Here, we report a long-read genome assembly for Saccharomyces uvarum strain CBS 7001 based on PacBio whole-genome shotgun sequence data. Our assembly provides an improved reference genome for an important yeast in the *Saccharomyces sensu stricto* clade.

## ANNOUNCEMENT

Saccharomyces uvarum is a globally distributed yeast species that can be commonly extracted from low-temperature fermentations and other natural substrates (1–6). Low-coverage Sanger sequence assemblies were initially reported for an S. uvarum reference strain called CBS 7001 (also called MCYC 623) (7, 8). Later, Scannell et al. (9) generated a chromosome-level scaffolded assembly for CBS 7001 that combined Sanger and Illumina short-read sequences. All previous CBS 7001 assemblies contain a large number of gaps (Table 1), limiting the comprehensive analysis of transposable elements (TEs) in this species (9, 10). Here, we generated a long-read genome assembly for strain CBS 7001 to improve the reference genome and support analysis of TEs in S. uvarum.

**TABLE 1:** Statistics for *de novo* assemblies of CBS 7001^*a*^

Assembly	NCBI accession no.	No. of contigs	No. of scaffolds	Total length (bp)	Contig *N*_50_ (bp)	Complete BUSCOs (%)	No. of full-length *Tsu4* copies
Kellis et al. (7)	GCA_000166995.1	1,098	1,098	11,475,890	4,795	88.3	0
Cliften et al. (8)	GCA_000167035.1	588	586	11,862,889	19,032	94.2	3
Scannell et al. (9)	NA^*b*^	363	16	11,502,913	18,172	97.1	0
This study	CM034478 to CM034493	17	16	11,968,998	836,618	97.3	9

a The contigs within a scaffold were defined by 10 consecutive Ns.

b NA, not applicable. The CBS 7001 genome assembly from Scannell et al. (9) is available at http://www.saccharomycessensustricto.org/.

To prepare DNA for PacBio sequencing, a single colony of strain CBS 7001 (NCYC 2909) was inoculated in 7 mL yeast extract-peptone-dextrose (YPD) liquid broth and cultured for ∼24 h at 30°C. DNA was isolated using the Wizard genomic DNA purification kit (Promega), and a PacBio library was prepared using the SMRTbell Express template prep kit (Pacific Biosciences), following the >15-kb size-selection protocol that includes Covaris g-TUBE shearing. PacBio sequencing was performed using the Sequel II instrument (sequencing kit v2.1), yielding 655,409 reads (average length, 8,490 bp). HGAP4 (smrtlink-release_5.1.0.26412) (11) was used for *de novo* assembly, and the resulting PacBio contigs were polished five times using Pilon v1.24 (12) with 33,377,822 51-bp Illumina Genome Analyzer (GA) II reads (SRA accession number SRR173084). The polished contigs were scaffolded using RagTag v2.0.1 (13) with CBS 7001 ultrascaffolds from reference 9 that corrected the name swap for ChrX and ChrXII noted in reference 5. The assembly statistics were calculated using stats.sh from BBMap v38.90 (14) and BUSCO v5.0.0 (saccharomycetes_odb10) (15). TEs were annotated using a RepeatMasker-based pipeline described in reference 16. Genome alignment was performed using MUMmer v3.23 (17). The PacBio reads were aligned to the genome assemblies using minimap2 v2.17 with the parameters “-ax map-pb” (18). Default software parameters were used except where otherwise noted.

Our CBS 7001 assembly is 11,968,998 bp long (contig *N*_50_, 836,618 bp) with an overall GC content of 40.18% (Table 1). Of the chromosomes, 15/16 are assembled in scaffolds with one contig each; ChrXII is assembled in one scaffold with one gap at the rDNA locus. In total, 97.3% of *Saccharomycetes* benchmarking universal single-copy orthologs (BUSCOs) are present and single copy. Our assembly was colinear with all scaffolds in the Scannell et al. (9) assembly except for one discrepancy at a sequencing gap in the previous assembly (ChrIII: 219401 to 219500). PacBio reads aligned to the previous assembly did not span this gap but did align continuously through the corresponding region in our assembly. Our assembly corrects this misassembly, the ChrX ↔ ChrXII name swap (5), and closes over 340 sequencing gaps in the previous assembly. Our assembly provides confirmation that *Tsu4* is the only TE with full-length copies in the CBS 7001 genome (19–22): we found nine full-length *Tsu4* copies in our assembly, tripling previous estimates (8, 23) (Table 1).

### Data availability.

The assembly produced here was deposited at NCBI under accession numbers CM034478 to CM034493. The PacBio data used to generate the assembly are available under SRA accession number PRJNA753102.
